# Neural Mechanisms of Self-Location

**DOI:** 10.1016/j.cub.2014.02.049

**Published:** 2014-04-14

**Authors:** C. Barry, N. Burgess

**Affiliations:** 1 UCL Research Department of Cell & Developmental Biology, Gower Street, London, WC1E 6BT, UK; 2 UCL Institute of Cognitive Neuroscience, London, WC1N 3AR, UK; 3 UCL Institute of Neurology, Queen Square, London, WC1N 3BG, UK

## Abstract

The ability to self-localise and to navigate to remembered goals in complex and changeable environments is crucial to the survival of many mobile species. Electrophysiological investigations of the mammalian hippocampus and associated brain structures have identified several classes of neurons which represent information about an organism’s position and orientation. These include place cells, grid cells, head direction cells, and boundary vector cells, as well as cells representing aspects of self-motion. Understanding how these neural representations are formed and updated from environmental sensory information and from information relating to self-motion is an important topic attracting considerable current interest. Here we review the computational mechanisms thought to underlie the formation of these different spatial representations, the interactions between them, and their use in guiding behaviour. These include some of the clearest examples of computational mechanisms of general interest to neuroscience, such as attractor dynamics, temporal coding and multi-modal integration. We also discuss the close relationships between computational modelling and experimental research which are driving progress in this area.

## Teaser

The ability to navigate in complex and changeable environments is crucial to the survival of many mobile species. Barry and Burgess review the computational mechanisms by which the spatial representations mediated by place, grid, head-direction and boundary-vector cells are formed and used to guide behavior, and discuss the close relationships between computational modelling and experimental research in this area.

## Main Text

### Introduction

The ability to self-localise — to determine one’s current position within the environment — is an essential process for humans, mammals in general, and many other mobile species. Indeed, being able to self-localise is a necessary requisite for successful navigation to any goal that is not directly detectable. The scientific literature on this topic is extensive, from Darwin, who speculated on the sources of information that animals draw on to self-localise [1], to modern robotic devices such as global positioning systems.

**Figure 1:**
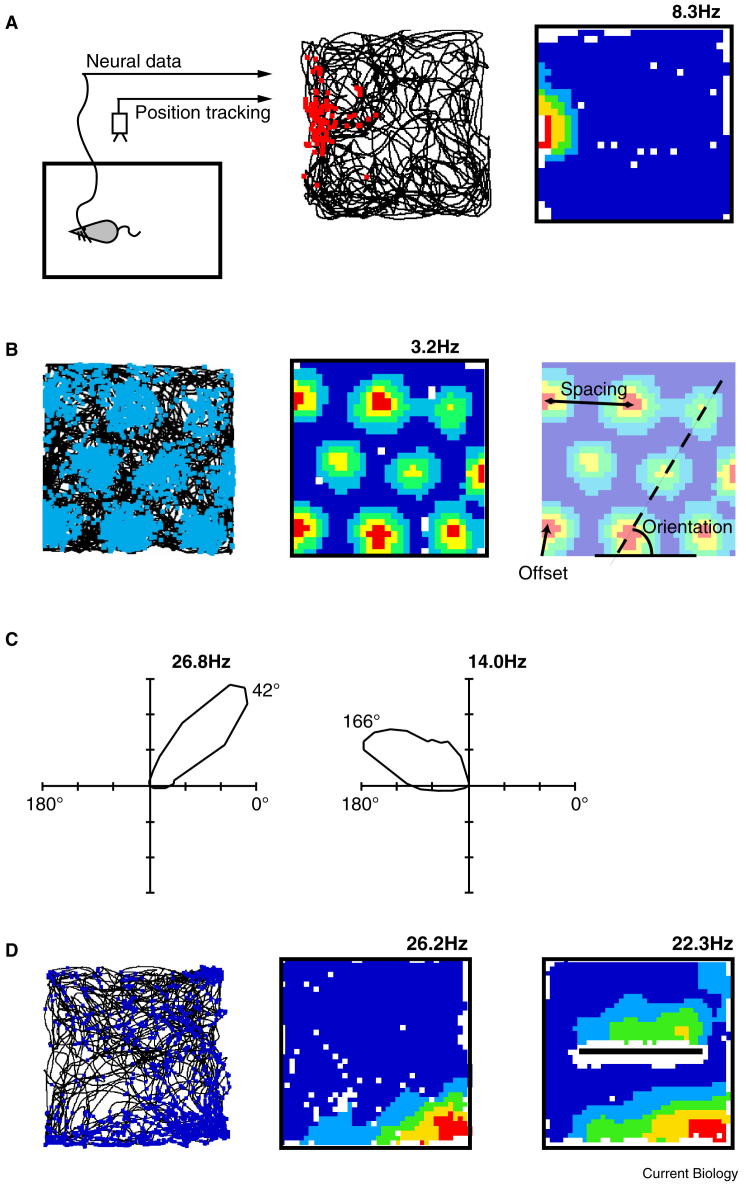
Neural representations of self-location in the hippocampal formation. (A) Left, schematic of single unit recording. A rodent with chronically implanted extracellular electrodes forages in an open environment, with surrounding sensory cues for orientation (not shown). Tracking data from an overhead camera are synchronized with neural data. Middle, raw data from a place cell. The animal’s path is indicated by the black line, and action potentials are superimposed in red at the locations where they were emitted. Right, a firing rate map of the raw data; binned spike count is divided by binned dwell time and locally smoothed to calculate average firing rate. ‘Hotter’ colours indicate higher firing rates reaching a maximum of 8.3 Hz (indicated above the map), dark blue indicates low rate (0 to 20% of the peak rate), white bins are unvisited. This CA1 place cell is only active when the animal occupies a small area on the west of the environment. (B) Raw data (left) and firing rate map (middle) for a mEC grid cell. The multiple circular firing fields are arranged in a close packed hexagonal lattice. Right, the regular grid-like firing pattern is characterised by its orientation, spacing, and offset. (C) Two head direction cells recorded from the deep layers of mEC; similar directional responses are exhibited by head direction cells found in other brain regions. The polar plots show firing rate as a function of head direction; the cell on the left has a peak firing rate of 26.8 Hz achieved when the animal was facing an orientation of 42° relative to the environment (measured anti-clockwise from the horizontal axis). (D) A boundary vector cell in the subiculum, showing the raw data (left) and firing rate map (middle). The boundary vector cell fires whenever there is an environmental boundary a short distance to the south. The boundary vector cell shows a second firing field after an east–west oriented barrier is put into the environment (right).

In recent decades, neurons have been identified in the mammalian brain the firing of which encodes information about the spatial location and orientation of the animal relative to its environment. These include place cells, which fire whenever the animal enters a specific location; head direction cells, which fire whenever the animal’s head is in a particular orientation; and grid cells, which fire whenever the animal enters any one of several locations arranged across the environment in a regular triangular array (Figure 1) [2–4]. Here, we briefly review the salient properties of these spatial representations, and then discuss the neural mechanisms that underlie their generation.

### Neuronal Representations of Environmental Location and Orientation

Extracellular recordings made in the 1970s from the hippocampi of freely moving rodents identified place cells in regions CA1 and CA3 [2]. Individual place cells are typically silent, only firing action potentials when the animal’s head is within a certain region of the environment — the cell’s place field (Figure 1A). The size and location of the place fields varies between place cells, providing a sparse population vector that carries sufficient information to represent the animal’s current location [5]. Initially identified in the rat, place cells have subsequently been found in animals as disparate as bats and humans [6,7] and are believed to be a common mammalian phenomenon.

When an animal enters a new environment for the first time, place cell firing patterns are established very rapidly [5]. In a familiar environment, place fields are stable: a cell will typically fire whenever the animal re-enters the firing field even after a delay of several days [8], although firing patterns may vary over these and longer durations [9–11]. Place cell firing patterns are environment-specific and have distinct firing patterns in different environments, changing their firing rates and firing locations relative to environmental features and each other, a process known as ‘remapping’ [12–14]. However, place cell activity is unaffected by subtle changes in a familiar environment; extinguishing the lights or eliminating a subset of cues, for example, does not generally affect spatial responses [15,16]. This process of ‘pattern completion’ and the related phenomena of ‘pattern separation’, whereby place cells disambiguate known environments despite their perceptual similarity [17], is indicative of attractor states. In other words, after small perturbations the network dynamics cause firing patterns to evolve back to specific stable states.

Marr’s influential model of hippocampal function was prescient of such attractor dynamics, identifying them with associative plasticity in the recurrent connections of area CA3 [18]. Consistent with his ideas, the place cells of mice without functional CA3 NMDA receptors (necessary for long-term potentiation of synaptic connections [19]) show impaired pattern completion, their firing being degraded as cues are removed from an environment [20]. The representation of self-location provided by place cells helps to guide spatial behaviour: cue manipulations that rotate place fields relative to the environment are matched by a concomitant rotation in the animal’s spatial responses. Even in error trials where the cells fail to follow the cues, behaviour generally covaries with place field location [21,22].

Subsequent to the discovery of place cells, investigation of related cortical and subcortical regions revealed complementary spatial responses. The first of these, head direction cells, signal the orientation of the animal’s head in the horizontal plane (azimuth): individual head direction cells respond when the animal occupies a narrow range of head directions (∼100°) centred on a preferred firing direction [23] (Figure 1C). These cells were first reported in the dorsal presubiculum [24,25] and later in a network of structures including the thalamic nuclei [26,27], mammillary bodies [28], and entorhinal cortex [29]. Unlike place fields, which change relative position between environments, the angular offset between the preferred firing directions of head direction cells is maintained across environments [30]. Thus, two cells that share a preferred firing direction in one environment will continue to respond at the same time in a second environment, even though the absolute firing direction of both cells may have changed. Like place cells, activity in the head direction system strongly correlates with behaviour. Hence, errors in the heading direction encoded by the cells are predictive of navigational errors [31]. Head direction cells are thus likely to be part of a network that provides an animal’s ‘internal sense of direction’.

The third main type of spatial cell to be found, grid cells, are most numerous in layer II of medial entorhinal cortex (mEC) and exhibit stable spatial firing correlates broadly similar to place fields [4]. Unlike place cells, however, they are characterised by multiple circular firing fields arranged in an equilateral triangular lattice across the environment (Figure 1B). In deeper layers of the mEC, as well as in the pre- and para-subiculum, grid cells co-localise with head direction cells and ‘conjunctive’ cells, which combine grid spatial firing with directional tuning [29,32].

Initially identified in rats, grid cells have since been found in other mammals including bats, mice, and humans [33–36]. In the mEC at least, they appear to be clustered into functional modules — the grid-like firing of neighbouring cells share the same orientation and scale, such that their spatial correlates are effectively translations of one another [4,37,38]. Like head direction cells, the relative position of the grid-like firing of cells from the same module is maintained even after manipulations that change or disrupt the fields of individual cells [38,39]. In contrast, cells from different modules seem to be more independent: their firing fields can respond differently to changes in the geometry of the environment [38]. Still there are some global organising principles: the orientation of grids in different modules is similar [37,38], and grid scale, which increases ventrally along the mEC [4], does so in discrete steps which may follow an approximately geometric series [37,38], with implications for optimal coding of self-location in large-scale space [40,41].

These electrophysiological findings provide powerful insights into the neural mechanisms supporting self-localisation. These insights have been quantified in the form of computational models that seek to explain the neuronal and behavioural data, and how each relates to the other. Intense interactions between computational and experimental approaches have been generated within this field of research, as predictions are tested and hypotheses revised.

An important theoretical distinction has been made between two potential sources of information supporting self-location: first, environmental information; and second, information reflecting self-motion. The former reflects sensory perception of the environment, including information regarding the locations of environmental features or landmarks around the animal. The latter includes information concerning self-motion from vestibular, proprioceptive, visual (optic flow) and motor (motor-efference copy) systems concerning the consequences or planning of self-motion. The two sources of information are complementary: environmental information gives direct information regarding location relative to the environment, whereas self-motion information can be used to update the estimate of environmental location (spatial updating), or to estimate the displacement caused by a recent movement (path integration). Other terms used for this dichotomy include allothetic versus idiothetic, and exteroceptive versus interoceptive, invoking a (partial) mapping onto sources of information that are external or internal to the body.

Below, we review the neural mechanisms supporting the spatial representations discussed above. First considering mechanisms focussed on environmental information, then on self-motion information, and finally on how the two types of information might work together to support accurate self-location.

### Neural Processing of Environmental Information

Animals use external landmarks to localise themselves and guide navigation [42,43]. For example, rats trained to find a reward on a four-arm maze do so with reference to the surrounding visual cues; if the cues are rotated relative to the maze, then the animals search in a location defined by the new cue position [21]. Non-visual cues, such as olfactory markings or auditory signals, also contribute to self-location and are sufficient to guide behaviour if visual cues are not available [44]. Similarly, simple manipulations of spatial cues also produce parametric changes in the firing fields of spatial cells. For example, in a circular arena polarised by a single cue card, the place cells and head direction cells are jointly oriented by the card [45,46], and the orientation of grid cell firing is also controlled in the same way [4]. However, if the cue card is removed the cells continue to respond — place fields, for example, maintain their position relative to each other but adopt a random orientation relative to the arena [45], as do head direction cells [25]. A complementary study, in which the size of the recording arena was varied without affecting orienting cues, found that individual place cells tended to respond at a fixed distances and allocentric directions from two or more of the arena’s walls [47] — that is, referenced relative to the world as opposed to the self (egocentric).

**Figure 2:**
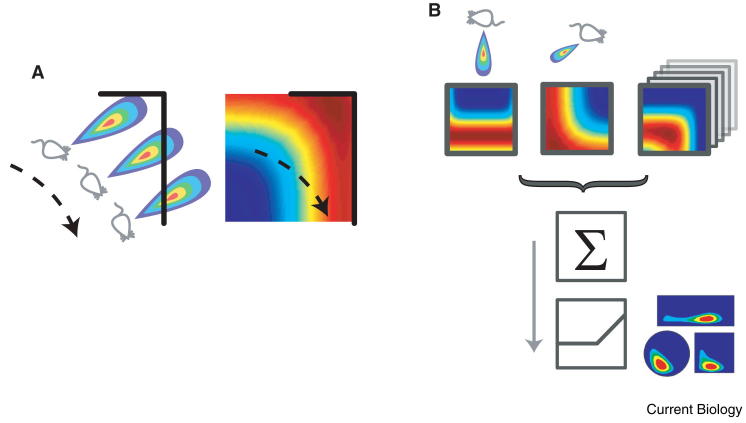
The boundary vector cell model of place cell firing. (A) A boundary vector cell responds when a boundary (black line) occupies its receptive field which is peaked at a preferred distance and allocentric direction from the animal (a short distance to the northeast in this example, left). This boundary vector cell will have a firing rate map with raised firing along the northeast boundary of the environment (right). (B) The activity of a place cell is modelled as the summed and thresholded activity of a population of heterogeneous boundary vector cells (with different preferred distance and direction tunings, above). The place cell’s firing (below) can be estimated for any geometric arrangement of environmental boundaries. (Adapted with permission from [61].)

The effects of environmental manipulations suggest that place cell firing can be modelled as the threshold sum of a population of neurons responding to environmental boundaries. Each of these ‘boundary vector’ cells signal the presence of a boundary at a specific allocentric direction and distance [48,49] (Figure 2). The model specifies that the directional tuning of boundary vector cells is determined relative to the animal’s head direction system. Thus, in a symmetrical environment, cue manipulations that cause a rotation in the responses of the head direction system will be matched by a rotation of the place cell population. Further empirical studies have confirmed that even when the environmental geometry is substantially changed, such as by adding or moving walls, the position of place fields can be predicted on the basis of their position relative to the surrounding walls [48,50]. The recently discovered mEC border cells and subicular boundary vector cells (Figure 1D) closely match the characteristics predicted for the putative boundary vector cells — elongated firing fields running parallel to, and at a specific allocentric direction from, environmental boundaries, and which maintain their firing characteristics between environments [50–52]. It is now known the border cells project to the hippocampus, and it seems likely their activity shapes the spatial responses of place cells [53].

The boundary vector cell model emphasises the importance of environmental boundaries in defining place cell activity without precisely specifying how they are perceived or tracked when not directly detectable, but see [49,54]. This high level approach followed a number of earlier models describing place field firing in terms of the feed-forward activity of sensory cells, such as those responding to visual ‘local views’ [55–57]. However, place cell firing is clearly influenced by multiple modalities of sensory input. For example, manipulations which move local cues (textured and coloured surfaces) relative to distal cues produced a heterogeneous response [58]: some place fields maintained their position relative to the local cues; some followed the visual distal cues; and others (∼40%) exhibited more complex responses, possibly indicative of joint influences [59].

Under the same conditions head direction cells always rotate coherently, normally following the distal cues [60]. Similarly, in a study in which arenas were distinguished on the basis of their colour and odour, place cell firing also distinguished the environments (‘remapped’), some on the basis of colour or odour alone, and others showing more complex conjunctive responses [14]. The boundary vector cell model does not account for these heterogeneous responses, at least not in its basic form; although more complex responses to environmental manipulations were obtained from a model in which boundary vector cells learned to respond differentially to different types of boundary [61]. It seems likely, however, that while boundary vector cell-type spatial responses influence place cell firing, these are themselves modulated by non-spatial inputs [14], which could potentially gate firing via an overall change in membrane potential [62]. Thus, if sufficient changes are made to an environment, the boundary vector cell input to a place cell will change significantly, producing a remapping of its place field.

### Neural Processing of Self-motion

Alongside the strong role for environmental information, reviewed above, animals can also self-localise in the absence of external sensory cues. For example, in the dark, gerbils are able to search for a missing pup and return with it directly to the nest [63]. This process, known as path integration or dead reckoning, requires the animal to update its representation of self-location based on the cumulative estimate of the distance and direction it has travelled [64]. It can be shown that an animal is utilising path integration by introducing a known error into its representation of direction or distance: in the case of the gerbils, if they are rotated prior to the return leg of the journey, and this is done slowly so that the vestibular system does not detect the motion, then the animals head towards the nest with an angular error equal to the amount they were rotated by [63].

By its nature, path integration is an iterative process and errors will accumulate unless corrected by reference to environmental information. This tendency to accumulate error limits the range over which path integration alone — both linear and angular — can support effective self-location. For example, in the dark, hamsters can make approximately three full circuits around the centre of an environment before they become too disorientated to travel directly to a nest at the periphery [65]. Interestingly, the rate of accumulation of error depends on the frame of reference within which self-motion information is integrated, with an advantage for allocentric over egocentric frames [66].

As with spatial behaviour, there is evidence that neuronal spatial representations are also influenced by self-motion information. For example, removing individual cues from the environment or extinguishing the lights often has little effect on spatial neuronal firing [4,15,16,21,67]. Furthermore, changes in place cell firing caused by environmental manipulations also reveal influences of self-motion. Expansion of the environment reveals the separable influences on place cell firing of the boundaries ahead of and behind the animal, but also an additional influence of the boundary that the animal is running away from, suggesting an additional role of self-motion coding [47,68,69]. More recently, virtual reality has allowed explicit demonstration of the influences of both environmental (visual) and self-motion (proprioception and motor-efference) information on the spatial firing of place cells [70].

While place cell firing has generally been recognised to reflect a balance between environmental and self-motion inputs [71,72], head direction cells and grid cells have been predominantly associated with the integration of self-motion information, with the subsequent addition of environmental information to correct the accumulation of error (but see [73]). The fixed relative offset maintained between the spatial responses of pairs of grid cells or pairs of head direction cells strongly hints at an endogenously generated mechanism, as does the regular periodic nature of grid cell firing. Two main classes of mechanism have been proposed to account for the way in which self-motion information influences neuronal spatial representations: continuous attractor models and oscillatory interference models, which we review below.

#### Continuous Attractor Networks

The head direction system has been understood in terms of continuous attractor networks. In these models, network activity is restricted to a limited state space, through which it can smoothly transition; population activity will relax back on to this manifold if it is perturbed away from it by an external influence. For the head direction system, this is the equivalent of the network only exhibiting firing patterns consistent with a single direction of facing at a given time, as appears to be the case [30,74].

**Figure 3:**
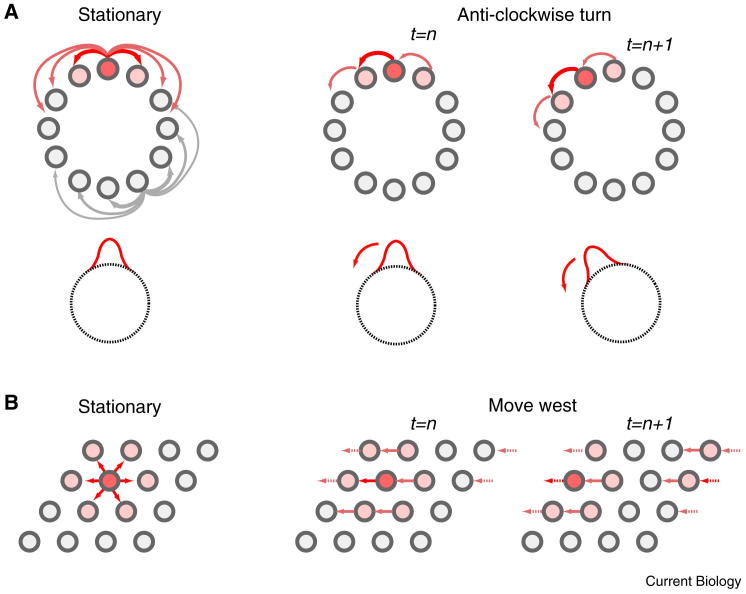
Schematic continuous attractor networks. (A) Left: the head direction system can be modelled as a circular continuous attractor network. Head direction cells (grey circles) are shown arranged according to their preferred firing direction, with raised firing rates indicated by warmer colours. Cells are reciprocally connected with their neighbours such that those with more similar preferred firing directions have stronger connectivity (schematic connections from the most active cell shown in red: thicker arrows indicating stronger connectivity). Connectivity is translation invariant such that all cells have similar connectivity profiles (connectivity for a second cell is shown in light grey). Activity in the head direction cell population will settle into a stable state, comprising a single activity ‘bump’, signalling the animal’s facing direction (schematic activity ‘bump’ shown in red, below). Middle and right: the activity bump can be moved around the circle by introducing an asymmetry into inter-cellular interactions. To track the animal’s heading, the strength of this asymmetric component must be proportional to the angular velocity of the animal’s head. This can be achieved by ‘shifter cells’ whose firing is modulated by head direction and angular velocity. Asymmetric connectivity producing an anti-clockwise rotation is shown (above) with the activity bump (below). (B) Left: populations of grid cells with grid-like firing patterns of the same orientation and scale can be modelled as a two-dimensional continuous attractor network: grid cells are shown arranged in a sheet according to the relative offset of their firing patterns. Cells are reciprocally connected with their neighbours so that those with closer firing patterns have stronger connectivity; this connectivity pattern is translations invariant across the sheet of cells. Again, a stable activity bump will form, signalling a static location. Right: the activity bump can be moved across the sheet of cells by asymmetric connectivity. To track the animal’s movement, the strength and direction of this asymmetrical component must be proportional to the animal’s velocity. This can be achieved by ‘shifter cells’ with grid-like firing that is also modulated by running velocity (that is, similar to conjunctive grid cells recorded from layers III–VI of the mEC [29]). The periodic nature of the spatial firing patterns corresponds to toroidal boundary conditions such that, as the activity bump moves off one side of the sheet, it appears on the other side.

Several continuous attractor models of the head direction system have been proposed (for example [75–78]), all sharing several key elements. First, cells have a graded profile of symmetrical inter-connectivity, so that the connection strength between two cells reflects the difference in their preferred firing directions, cells with similar preferred firing directions having stronger (more excitatory) connections than those with different preferred firing directions (Figure 3A). The connectivity profile, as a function of preferred firing direction, is translation invariant; this prevents inherent biases for particular directions, produces similarly shaped tuning curves relating firing to head direction for all cells (translated to reflect a cell’s preferred direction), and fixes the relative offset between the preferred directions of arbitrary cell pairs, as is observed experimentally [30,74]. With the cells arranged in a ring, each positioned according to its preferred firing direction, the pattern of activity will resemble a smooth bump, the location of which represents the animal’s head-direction. The bump of activity can move smoothly around the ring, with all represented directions equally likely.

For the population activity in the continuous attractor network to track the animal’s orientation, an asymmetry must be introduced to shift the bump of activity around the network as the animal turns [75]. This can be achieved by using a network of ‘shifter cells’ with asymmetric connectivity and firing modulated by both heading and angular velocity [77,78] (Figure 3A). Candidate shifter cells, with directional firing modulated by turning speed, have been identified in the anterior thalamus [26,79], presubiculum [80] and retrosplenial cortex [81]. Any such angular path integration mechanism will accumulate error, and must be corrected by reference to environmental cues, requiring the relationship between head direction activity and environmental sensory input to be learnt. This could be accomplished by Hebbian plasticity between visual feature detectors and head direction cells [75,77], and would be consistent with the observation that visual cues control the orientation of head direction cell firing [25]. In addition, the connection patterns in continuous attractor networks require precise calibration, which may be provided by reference to environmental inputs [75,77] or by angular velocity inputs [82].

Continuous attractor networks have also been used to model place and grid cell firing, extending the one-dimensional model of the head direction system to two dimensions. These require the same basic features as the one-dimensional models: translation invariant connectivity arranged so that cells with proximate fields are more strongly interconnected than those with distant fields, along with shifter cells, or some other form of tuneable asymmetric connectivity, to move the activity profile (Figure 3B). Indeed, before the discovery of grid cells, models of path integration focused on the possibility that the recurrent architecture of CA3 supported a continuous attractor network capable of updating place cell representation according to self-motion (for example [83–85]).

Place cells are less obviously compatible with the necessary network architecture than head-direction cells. Place cell remapping between environments requires different connectivity patterns in different environments, perhaps existing as multiple pre-configured ‘charts’ [84]. There is limited evidence for spatial shifter cells, which would be expected to be more numerous than the place cells themselves. Finally, there are many examples of heterogeneous changes in place cell firing — for example, where sub-populations of place fields change position or rate, sometimes in concert, while concurrently other cells are stable (for example [9,10,14,58,86]). Heterogeneity in this form would be inconsistent with a continuous attractor network, although there are some signs that CA3 place cells respond more homogenously than those in CA1 [12,59].

Grid cell firing, in contrast, shows many of the characteristics of a continuous attractor network. The firing patterns of neighbouring grid cells are often simple translations of each other [4] and their relative offsets remain fixed despite large changes to environmental conditions [39]. Conjunctive grid cells, whose grid-like firing patterns are also modulated by head direction, might plausibly function as shifter cells [29]. Finally, the fact that grid scale is discretised into multiple functional modules points to the presence of several distinct attractor networks, each corresponding to a single grid scale [37,38]. There is, however, as yet no direct evidence that grid cell firing patterns perform path integration in the way envisaged by continuous attractor network models.

#### Theta Oscillations and Self-Motion

**Figure 4:**
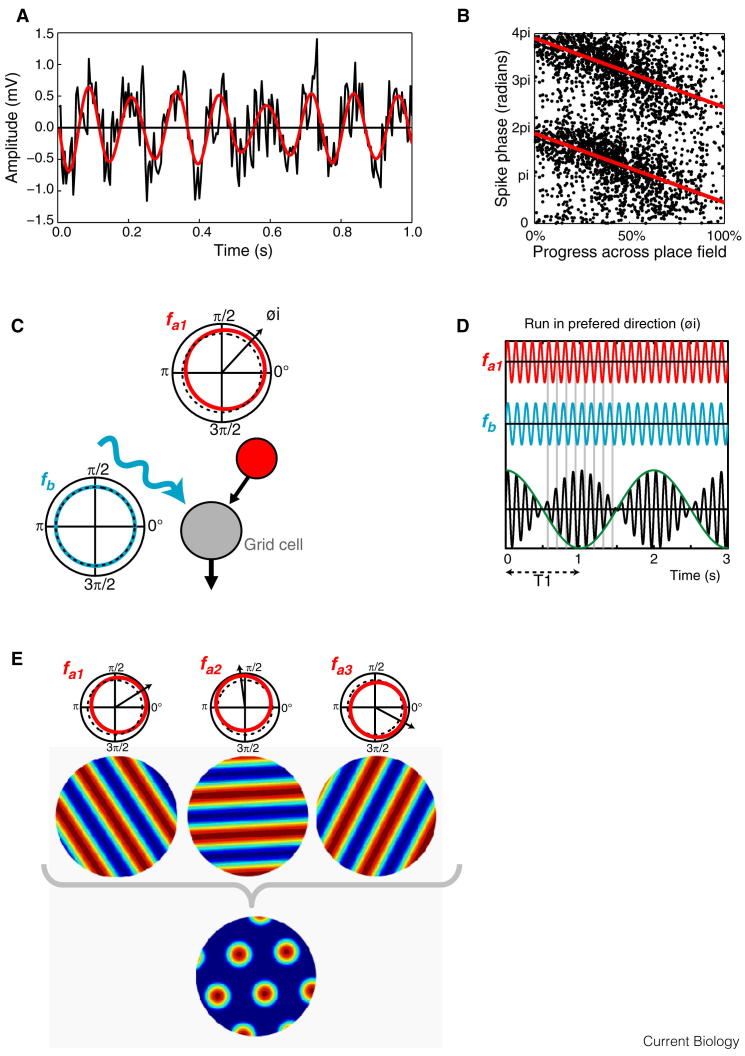
Theta-band oscillations structure spatial activity in the hippocampal formation. (A) Local field potential (LFP) recorded from the CA1 pyramidal cell layer of a moving rat. Black trace, raw mean-normalised LFP, in which the 8 Hz theta modulation is visible along with higher frequency gamma oscillations (the signal band-pass filtered in the 6–12 Hz range is shown in red). (B) Theta phase precession in a CA1 place cell: each point indicates, for a single action potential, the theta phase (y axis) and animal’s location (x axis); data from multiple runs through the place field, moving left to right. Red line indicates the circular-linear regression of phase on position. (C) Schematic of the oscillatory interference model showing two components: a baseline oscillation (blue, with frequency *fb*) and a velocity controlled oscillator (red) whose frequency (*fa1*) varies from baseline proportionate to the animal’s running speed in direction *φi*. (D) Interference pattern generated between the active and baseline oscillations in (C). Spikes are emitted at the peaks of the carrier (black) which is the sum of the two oscillations, showing a repeating periodic pattern. (E) If velocity controlled oscillator frequency *fa* varies around the baseline frequency with the animal’s movement proportional to the speed and the cosine of direction relative to a preferred direction (radial black arrow), then the baseline and velocity controlled oscillator sum to produce a spatially stable striped pattern perpendicular to the preferred direction. Multiple velocity controlled oscillators with preferred firing directions selected to differ by multiples of 60° produce a grid-like firing pattern. (Adapted with permission from [116].)

In parallel to the attractor models, a second stream of research has focussed on the movement-related theta rhythm: a 4–10 Hz oscillation that dominates the hippocampal local field potential (LFP) of moving rodents [87] and modulates the firing of place cells and many grid cells (Figure 4A). Theta frequency usually increases with running speed [88,89]. However, the theta-band modulation of firing of place and grid cells exceeds the LFP frequency, so that spikes are emitted at increasingly earlier phase of the LFP theta cycle as the animal moves through the firing field; an effect known as phase precession [90,91] (Figure 4B). Thus, the theta phase of firing encodes the distance travelled through the firing field, adding additional information on self-location beyond that encoded by firing rate alone [92].

Oscillatory interference models build upon the observation of phase precession by assuming the existence of ‘velocity controlled oscillators’, the frequency of which is modulated by the animal’s movement (Figure 4C). Specifically, their frequency varies around some baseline value proportional to the component of velocity along a preferred direction, so that their phase relative to baseline encodes displacement along that direction [93,94] (Figure 4D). Multiple velocity controlled oscillators, with different preferred directions, can form the basis of a path integration mechanism, by tracking displacement along multiple directions. They could also form the inputs to grid cells. In this case, velocity controlled oscillator inputs with preferred directions at multiples of 60°, as could be selected by unsupervised Hebbian learning during development [93,95], would sum together to produce the characteristic grid-like firing patterns (Figure 4E).

Cells resembling velocity controlled oscillators have been found in the anterior thalamic nuclei, medial septum and hippocampus; in these cells, the frequency of the theta-band modulation of firing varies as the component of velocity along a preferred direction [96]. Similarly, grid cell firing appears to be dependent on theta-band oscillations, as inactivation of the medial septum and consequent reduction of hippocampal and entorhinal theta are accompanied by a corresponding reduction of grid-like firing [97,98]. Finally, these models link the frequency of theta-band oscillations, at the cellular and LFP levels, with grid scale. In line with this prediction, factors that affect the frequency of theta-band oscillations, such as dorsoventral location, environmental novelty and deletion of the HCN1 channel subunit, are known to be accompanied by a concomitant change in grid scale [99–101].

In opposition to oscillatory interference models, grid-like firing is seen in crawling bats in the absence of theta rhythmicity [33], suggesting that grid firing does not always require theta. Nevertheless, it is possible that the very low firing rates of these cells obscures rhythmicity [102], or that baseline frequencies in these animals are lower than in rodents, as the absolute value of the baseline frequency is unrelated to the resultant spatial firing properties [94]. Indeed, the baseline frequency can even be zero, in which case velocity controlled oscillators equate to (non-oscillating) stripe-cells, which nonetheless sum to form grids [95]. In humans, theta rhythmicity has been linked to navigation [103] and memory [104], but how this corresponds with findings in other species remains controversial. Interference models have also been questioned by intracellular recordings made from mice exploring virtual reality environments; as expected, the phase of grid membrane potential oscillations was found to covary with position, but simple depolarisation of the cell was shown to account for more of the variability in spiking [105,106].

Interestingly then, both continuous attractor and oscillatory interference models enjoy some experimental support, while neither is clearly favoured. While these two types of model depend upon quite different mechanisms, they both describe grid cell firing in terms of path integrative input and are not incompatible. Indeed, hybrid models which incorporate recurrent connectivity and oscillatory dynamics have recently been proposed [107–109], and potentially provide a more complete account of the experimental data, explaining both the ramp depolarisation and membrane potential oscillation seen within the firing field [105,106,109].

### Combining Environmental and Self-Motion Information

An optimal estimate of self-location should combine both environmental and self-motion information. Indeed there is evidence that adult humans do use both types of information in a Bayes-optimal manner; each source being weighted by its reliability [110]. In rodents too, different spatial cues appear to be combined to guide behaviour, with visual cues generally being the most influential [44]. A similar pattern can be observed in terms of the orientation of place cell responses [111]. Furthermore, the combined influences of environmental and self-motion information on place cell firing can be seen in experiments in which a familiar environment is expanded [47,68]. Firing fields often become stretched or bi-modal along the expanded dimension in this situation, with sub-fields maintaining fixed distances from the boundaries ahead of and behind the animal, consistent with (environmental) boundary vector cell inputs. However, firing corresponding to the most recently visited boundary appears to have greater influence, potentially reflecting self-motion inputs or representational momentum within an attractor network [69]. Comparable environmental manipulations also show evidence of joint control of grid cell firing — if the size of a familiar environment is changed, grid firing initially ‘rescales’ commensurately, as if entirely driven by environmental inputs. However, with repeated environmental rescaling, the effect attenuates until the grid-like pattern no longer changes scale [37], as if simply representing a metric for self-motion.

The use of ‘virtual reality’ in rodent experiments, in which the animal runs on a polystyrene ball, which drives the viewpoint of visual projection onto a surrounding screen [112], has allowed for a more precise interrogation of the cues defining spatial responses. Although precluding vestibular inputs, virtual reality is sufficient to enable similar place cell [70] and grid cell [105] firing patterns compared to real environments. In this situation, most place cells (75%) required both visual and proprioceptive inputs to generate localised firing. For half of the place cells, presentation of the visual environment at the start of a run allowed place cells to fire in the correct location further along the track in terms of distance run on the ball.

**Figure 5:**
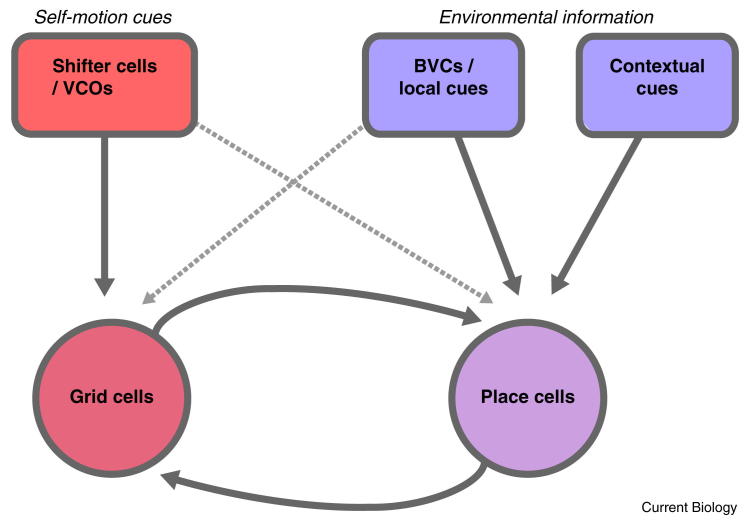
Potential arrangements of self-motion and environmental inputs to grid cells and place cells. The neural representations of self-location expressed by place and grid cells reflect both environmental and self-motion information. Grid cell activity (as well as head direction cell activity, not shown) is strongly influenced by self-motion cues which are proposed to originate from conjunctive shifter cells or velocity controlled oscillators. Place cells are strongly influenced by environmental information, including that relating to environmental boundaries (boundary vector cells), and to local cues (potentially from lateral entorhinal cortex) as well as non-spatial ‘contextual’ inputs that may ‘gate’ spatial inputs [14]. Regions containing grid cells and place cells are reciprocally connected, which may allow both representations to reflect an optimal combination of self-motion and environmental information [72]. Alternatively, self-motion and environmental information may respectively reach place cells and grid cells directly (dashed lines).

Thus, it seems likely that place and grid cells estimate self-location on the basis of both environmental and self-motion information. It is possible that environmental inputs, such as boundary vector cells, drive place cell firing, while grid cells are driven by self-motion inputs, and that unsupervised Hebbian learning between the two representations allows both types of information to be combined within a familiar environment (reviewed in [113]; Figure 5). This could explain the early development of stable place cell firing prior to stable grid cell firing [114] (but see also [115]) as well as many of the results described above (for example, grid cell rescaling, update of place cells in the dark).

### Conclusion

The neural representation of self-location is distributed across a network of brain regions and cell types, each encoding different elements of the spatial signal. Our understanding of how these populations interact to generate and update the observed spatial representations has benefited from a close relationship between empirical and theoretical work; the former providing the raw data to refine or contradict hypotheses suggested by the latter. In particular, competing predictions regarding place and grid cell firing, which could result from continuous attractor networks, or oscillatory interference (grid cells) or environmental inputs (place cells), have encouraged much experimental activity. We now have working models of potential neural mechanisms by which environmental cues can be combined with self-motion information to generate stable spatial representations.

Questions remain, though; for example, whether oscillatory activity and recurrent connectivity indicate incompatible or complementary mechanisms, and whether the relative influences of environmental and self-motion cues are dynamically reweighted according to their perceived reliability. More generally, models have been most successful when applied to neural systems with simple, constrained dynamics like the head direction, grid, and place cell responses in familiar environments. By contrast, the complex dynamics of place cell remapping between environments, which is believed to be a central component of memory formation, has proved to be less easy to predict.
